# Effect of EARLY administration of DEXamethasone in patients with COVID-19 pneumonia without acute hypoxemic respiratory failure and risk of development of acute respiratory distress syndrome (EARLY-DEX COVID-19): study protocol for a randomized controlled trial

**DOI:** 10.1186/s13063-022-06722-x

**Published:** 2022-09-15

**Authors:** Anabel Franco-Moreno, María Soledad Acedo-Gutiérrez, Nicolás Labrador-San Martín, Clara Hernández-Blanco, Celia Rodríguez-Olleros, Fátima Ibáñez-Estéllez, Ana Suárez-Simón, Mateo Balado-Rico, Ana Rocío Romero-Paternina, David Alonso-Menchén, Belén Escolano-Fernández, Esther Piniella-Ruiz, Ester Alonso-Monge, Helena Notario-Leo, Carlos Bibiano-Guillén, Gabriela Peña-Lillo, Armando Antiqueira-Pérez, Rodolfo Romero-Pareja, Noemí Cabello-Clotet, Vicente Estrada-Pérez, Jesús Troya-García, María de Carranza-López, Ismael Escobar-Rodríguez, Nacho Vallejo-Maroto, Juan Torres-Macho

**Affiliations:** 1 Department of Internal Medicine, Infanta Leonor–Virgen de la Torre University Hospital, Gran Via del Este Avenue, 80, 28031 Madrid, Spain; 2 Enfermera Isabel Zendal Emergency Hospital, Madrid, Spain; 3 Department of Internal Medicine, Puerta de Hierro University Hospital, Madrid, Spain; 4 Emergency Department, Infanta Leonor–Virgen de la Torre University Hospital, Madrid, Spain; 5 Department of Internal Medicine, Infectious Diseases Department, Clínico San Carlos University Hospital, Madrid, Spain; 6 Department of Pharmacology, Infanta Leonor–Virgen de la Torre University Hospital, Madrid, Spain; 7 Emergencia COVID-19 Hospital, Sevilla, Spain

**Keywords:** Adult respiratory distress syndrome, Corticosteroids, COVID-19 pneumonia, Dexamethasone, Inflammatory biological markers, Laboratory markers, Mortality, Randomized controlled trial

## Abstract

**Background:**

Corticosteroids are one of the few drugs that have shown a reduction in mortality in coronavirus disease 2019 (COVID-19). In the RECOVERY trial, the use of dexamethasone reduced 28-day mortality compared to standard care in hospitalized patients with suspected or confirmed COVID-19 requiring supplemental oxygen or invasive mechanical ventilation. Evidence has shown that 30% of COVID-19 patients with mild symptoms at presentation will progress to acute respiratory distress syndrome (ARDS), particularly patients in whom laboratory inflammatory biomarkers associated with COVID-19 disease progression are detected. We postulated that dexamethasone treatment in hospitalized patients with COVID-19 pneumonia without additional oxygen requirements and at risk of progressing to severe disease might lead to a decrease in the development of ARDS and thereby reduce death.

**Methods/design:**

This is a multicenter, randomized, controlled, parallel, open-label trial testing dexamethasone in 252 adult patients with COVID-19 pneumonia who do not require supplementary oxygen on admission but are at risk factors for the development of ARDS. Risk for the development of ARDS is defined as levels of lactate dehydrogenase > 245 U/L, C-reactive protein > 100 mg/L, and lymphocyte count of < 0.80 × 10^9^/L. Eligible patients will be randomly assigned to receive either dexamethasone or standard of care. Patients in the dexamethasone group will receive a dose of 6 mg once daily during 7 days. The primary outcome is a composite of the development of moderate or more severe ARDS and all-cause mortality during the 30-day period following enrolment.

**Discussion:**

If our hypothesis is correct, the results of this study will provide additional insights into the management and progression of this specific subpopulation of patients with COVID-19 pneumonia without additional oxygen requirements and at risk of progressing to severe disease.

**Trial registration:**

ClinicalTrials.gov NCT04836780. Registered on 8 April 2021 as EARLY-DEX COVID-19.

**Supplementary Information:**

The online version contains supplementary material available at 10.1186/s13063-022-06722-x.

## Background

Patients with coronavirus disease 2019 (COVID-19) infection are at risk of developing pneumonia, acute respiratory distress syndrome (ARDS), multi-organ failure, septic shock, and death. ARDS is a consequence of an alveolar injury producing diffuse alveolar damage. Cytokine storm is involved in the development of ARDS in COVID-19 patients [1]. This cytokine storm in COVID-19 is characterized by an excessive inflammatory response to SARS-CoV-2 caused by a dysregulated immune system of the host. Around 30% of COVID-19 patients with mild symptoms at presentation will progress to severe life-threatening disease largely due to ARDS [2]. Early control of the cytokine storm through therapies is essential to prevent mortality in patients with COVID-19 [3–5].

Corticosteroids such as dexamethasone inhibit the host’s excessive inflammatory response via its anti-inflammatory properties. Based on the Guidelines of the Society of Critical Care Medicine/European Society of Intensive Care Medicine (SCCM/ESICM), the use of systemic glucocorticoid therapy in ARDS is recommended for patients who are relatively early in the disease course (within 14 days of onset) and who have persistent or refractory moderate to severe ARDS (partial arterial pressure of oxygen/fraction of inspired oxygen [PaO2/FiO2] ratio < 200), despite initial management with standard therapies, including low tidal volume ventilation [6]. Corticosteroid treatment in ARDS was associated with a significant reduction in biomarkers of systemic inflammation, reduction in the duration of mechanical ventilation, and reduction in-hospital mortality [7–10]. In addition, the disease resolution is faster with early initiation of glucocorticoid (< 7 days) compared with late initiation.

Current COVID-19 Treatment Guidelines recommend the use of dexamethasone in patients who require respiratory support [11–14]. Results from the Randomized Evaluation of COVid-19 thERapY (RECOVERY) trial demonstrated a reduction in 28-day mortality with 6 mg daily of dexamethasone for up to 10 days compared to standard of care in patients hospitalized with COVID-19 who were receiving either invasive mechanical ventilation or oxygen alone at randomization [15]. Patients without respiratory support needs did not seem to benefit from corticosteroid use. Similarly, in a prospective meta-analysis that pooled data from seven randomized clinical trials of critically ill patients with COVID-19, systemic corticosteroid administration was associated with lower 28-day all-cause mortality compared with usual care or placebo [16].

An increase in inflammatory biomarkers is known to correlate with progression to ARDS and mortality in COVID-19 patients. Available data provide evidence for the differentiation of severe and non-severe cases of COVID-19 based on inflammatory biomarkers including lactate dehydrogenase (LDH), C-reactive protein (CRP), and lymphocyte count [17, 18]. Elevated LDH increase mortality (OR, 4.22; 95% confidence interval [95% CI], 2.49–7.14) in patients with COVID-19 [19]. For each 100-unit increase in CRP levels, the odds of death increase twofold (OR, 2.01; 95% CI, 1.57–2.56) [20]. Finally, a lymphocyte count lower than 0.95 × 10^9^/L is associated with a risk of death compared to patients with lymphocyte counts greater than 0.95 × 10^9^/L (OR, 7.27; 95% CI, 1.10–48.25) [21].

As mentioned above, in the RECOVERY trial, dexamethasone did not reduce mortality in patients with SARS-CoV-2 pneumonia without the need for supplemental oxygen. Nevertheless, we postulate that the use of corticosteroids in COVID-19 pneumonia in its initial phases might have a different effect in patients with inflammatory biomarkers associated with disease progression. One-third of COVID-19 patients with mild symptoms at presentation will develop severe illness, particularly patients in whom biomarkers associated with COVID-19 disease progression are detected. Therefore, we conducted a study to evaluate whether the early administration of dexamethasone in hospitalized patients with COVID-19 pneumonia without additional oxygen requirements in whom biomarkers with a strong correlate with severe prognosis are detected, leads to a decrease in the development of ARDS and death.

## Methods/design

### Justification of the study

Currently, dexamethasone is one of the few drugs that has shown a reduction in mortality in COVID-19 patients [15]. The RECOVERY trial found no benefit in patients with COVID-19 who did not require supplementary oxygen. Unfortunately, the RECOVERY trial did not collect laboratory biomarkers to predict the risk of the development of ARDS in these patients. Hence, the need for this study is based on the possibility that the benefit of dexamethasone in this specific subpopulation of patients was underestimated. Our goal in this study is to analyse dexamethasone’s efficacy in preventing the development of ARDS and death in patients with COVID-19 pneumonia who do not require respiratory support, and in whom biomarkers independently associated with increased risk of severe disease are detected.

### Study design

Effect of EARLY administration of DEXamethasone in patients with COVID-19 pneumonia without acute hypoxemic respiratory failure and risk of development of acute respiratory distress syndrome (EARLY-DEX COVID-19) study is a multicenter, randomized, controlled, open-label, parallel-group trial involving 252 hospitalized patients with COVID-19 pneumonia who do not require supplemental oxygen on admission, and laboratory biomarkers related to severe COVID-19 disease are detected. Patients will be randomized and enrolled in Spain. Study sites are listed in the Appendix.

The trial was designed in accordance with the Declaration of Helsinki [22], the Convention of the European Council related to human rights and biomedicine, and within the requirements established by Spanish legislation in the field of biomedical research, the protection of personal data, and bioethics, which was registered on 8 April 2021 at http://www.clinicaltrials.gov with identification no. NCT04836780. The study protocol (version 1.2, 17 April 2021) was approved by the Ethics Committee for investigation with medicinal products of the Comunidad de Madrid, Spain, and the institutional review boards of all participating hospitals (Additional file 1). The trial was approved by the Spanish Agency of Drugs and Medical Devices as a clinical randomized study with drugs on 25 May 2021 (Additional file 2 in Spanish).

Prior to any activity carried out as part of the research, subjects will receive a concise and focused presentation of key information about the clinical trial, verbally and with a written consent form. To be included, written informed consent will be requested from patients’ relatives or legal representative by the local investigators. Following the recommendations of the Spanish Agency of Medicines and Medical Devices, during the COVID-19 pandemic, patient consent can be obtained orally and preferably before an impartial witness, documenting it in the patient’s medical records [23]. The forms have been reviewed by the Ethics Committee that authorized the trial.

Our protocol followed the SPIRIT (Standard Protocol Items: Recommendations for Interventional Trials) guidelines [24]. See Additional file 3 for the SPIRIT checklist of the study protocol.

### Inclusion criteria

To be eligible for inclusion, patients must fulfil the following criteria during screening and prior to enrolment: hospitalized patients aged 18 years or older with confirmed SARS-CoV-2 pneumonia who do not require supplemental oxygen, and risk of developing ARDS. The risk of ARDS is defined by the presence of at least two of the following inflammatory biomarkers: LDH > 245 U/L, CRP > 100 mg/L, and lymphocyte count < 0.80 × 10^9^/L (Table 1).

**Table 1:** Inclusion criteria

a) Adults (age 18 years or older)
b) Confirmed COVID-19 based on a positive RT-PCR test or rapid antigen test for SARS-CoV-2 RNA in a respiratory specimen (nasopharyngeal or nasal swab)
c) Requiring in-hospital care
d) A chest imaging study compatible with pneumonia (X-ray or computed tomography)
e) SpO2 ≥ 94% and < 22 bpm breathing on room air
f) The presence of at least two of the following inflammatory biomarkers:
• LDH > 245 U/L
• CRP > 100 mg/L
• Lymphocyte count < 0.80 × 10^9^/L

*Abbreviations: SpO2* Peripheral capillary oxygen saturation, *bpm* Breaths per minute, *LDH* Lactate dehydrogenase, *CRP* C-reactive protein

### Exclusion criteria

The exclusion criteria include one or more of the following: patients with a history of allergy to corticosteroids, need for supplementary oxygen, pregnancy or breastfeeding status, oral or inhaled corticosteroids treatment in the preceding 15 days, human cytochrome P450 (CYP) enzyme–inhibiting drug treatment, use of immunosuppressants or immune-modulators (including chemotherapy) in the preceding 30 days, neutropenia < 1.0 × 10^9^/L, human immunodeficiency virus infection with CD4 cell counts < 0.50 × 10^9^/L in the preceding 90 days, dementia, liver disease defined as alanine aminotransferase (ALT) or aspartate aminotransferase (AST) ≥ 5 times the upper limit of normal, chronic kidney disease defined as a glomerular filtration rate ≤ 30 mL/min, haemodialysis or peritoneal dialysis, uncontrolled infection, and participation in clinical trials of any kind in the previous 28 days.

### Randomization

Eligible, consenting patients will be randomly assigned in a 1:1 ratio to receive either dexamethasone plus standard of care (intervention group) or standard of care alone (control group). Randomization will be done within the REDCap system with unpredictability of assignments. Our statistician created a randomization schedule using a permuted block design, so equal numbers are assigned to each group. This system ensures a balanced allocation of subjects to the study arms. Records of individual assignments to each group will be de-identified (user number and centre ID instead of patients’ names), sequentially numbered, and stored on a secure computer system. Local investigators in participating centres are the only authorized personnel to interact with the radomization system through a username and password. Once eligibility is confirmed in the electronic case report form, the treatment group is assigned by the system. Dexamethasone will be provided free of charge for this study by the organization funding. According to the ethical principles for medical research of the Declaration of Helsinki, the use of no placebo (no intervention) is acceptable when no proven intervention exists. The Spanish Agency of Drugs and Medical Devices and the Ethics Committees did not mandate a blinded design or the administration of a placebo.

### Intervention

**Fig. 1:**
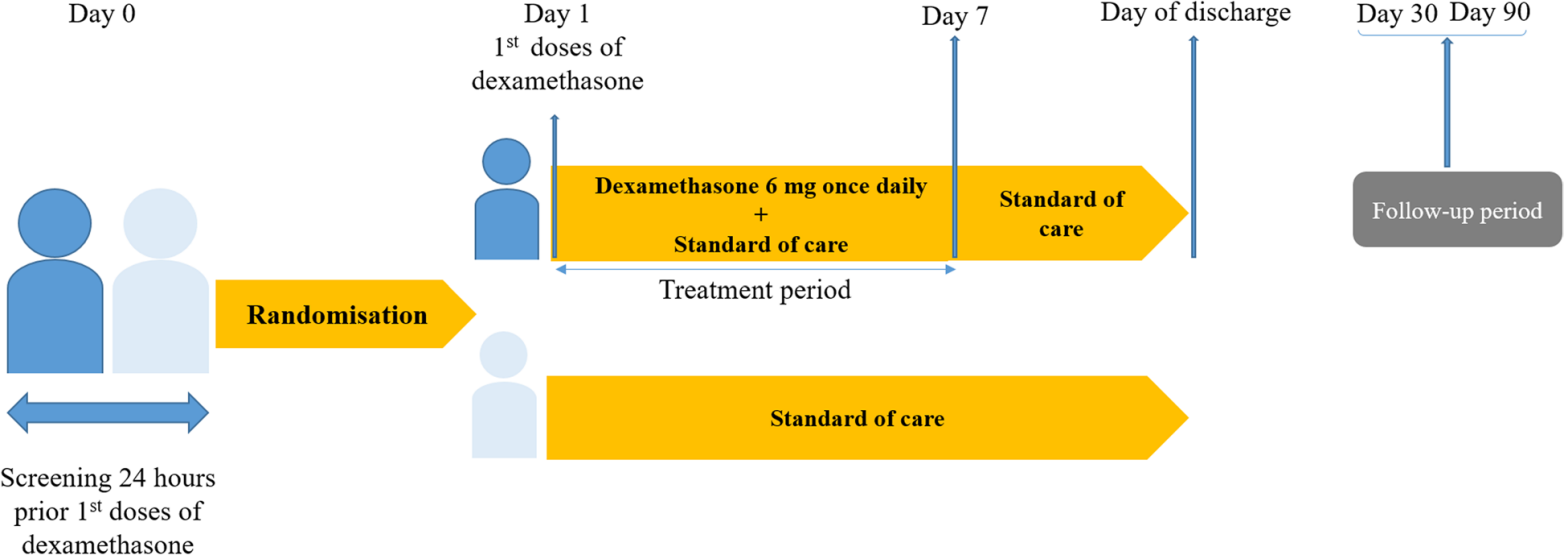
Study design diagram

Patients in the intervention group will receive an intravenous dose of 6 mg once daily from day 1 to day 3, followed by an oral dose of 6 mg once daily from day 4 to day 7 (Fig. 1). For participants unable to take an oral drug, intravenous dexamethasone will be given. We selected the same dose as the RECOVERY trial. Participants randomized to the control group will receive a standard of care. Given that pulmonary disease can progress rapidly in patients with COVID-19, patients will be closely monitored. For improving intervention adherence, a real-time medication monitoring system will offer monitoring of participants’ medication control combined with a short message service (SMS) after discharge. Study participants will receive the best standard of care according to the current COVID-19 Treatment Guidelines. Hospitalized patients who do not require supplemental oxygen can receive anticoagulants for the prevention of venous thromboembolic disease, gastric ulcer prophylaxis, hydration, antipyretics, antibiotics, and bronchodilators. Corticosteroids in patients requiring oxygen support in the control group will be received. In addition, remdesivir, tocilizumab, baricitinib, tofacitinib, and sarilumab will be considered in case of a worsening of disease status [13]. All chronic medication prescribed to the patient is permitted to continue at the discretion of the responsible physician/investigator.

For a trial participant, the assigned study intervention will be modified or discontinued by trial investigators if serious adverse events occur during the study period or withdrawal of participant consent. Regardless of any decision to modify or discontinue their assigned intervention, study participants will be retained in the trial whenever possible to enable follow-up data collection and prevent missing data.

### Data collection and procedures study

All participating patients, regardless of the study arm into which they are randomized, will be monitored following general standard of care practices. Patients will be assessed once daily by local investigators.

Demographic data including age and sex assigned at birth, comorbidities, and the COVID-19 vaccination status from participants will be collected. Temperature, respiratory rate, cardiac frequency, peripheral capillary oxygen saturation (SpO2), respiratory support, partial pressure of arterial oxygen/fraction of inspired oxygen (PaO2/FiO2), quick Sequential Organs Failure Assessment (qSOFA) score, and the ordinal scale for clinical improvement of the World Health Organization will be monitored at least once daily. Biochemistry and haematological tests on days 1 and 7; and chest X-ray on days 1, 4, and 7; and discharge will be assessed (Table 2). Laboratory determinations will include haemoglobin, white blood cell, lymphocyte count, platelets count, glucose, blood urea nitrogen, serum creatinine, sodium, potassium, LDH, alkaline phosphatase, albumin, ALT, AST, total bilirubin, CRP, prothrombin time, D-dimer, ferritin level, interleukin-6, and procalcitonin. Patients will be followed up to 90 days after randomization.

**Table 2:**
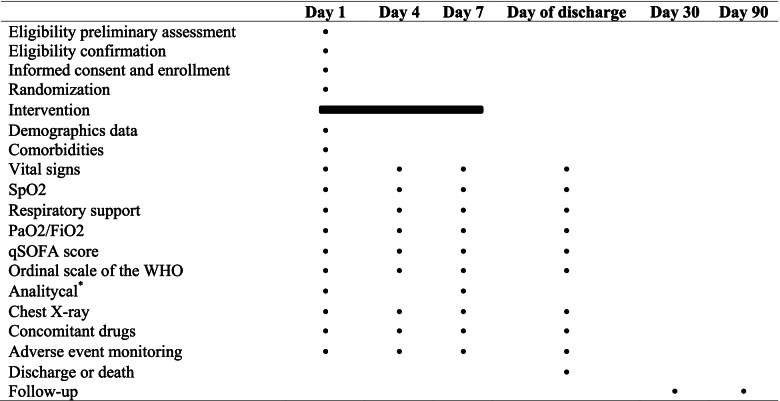
Scheduled protocol activities

*Abbreviations: SpO2* Peripheral capillary oxygen saturation, *PaO2* Partial pressure of arterial oxygen, *FiO2* Fraction of inspired oxygen, *qSOFA* Quick Sequential Organs Failure Assessment, *WHO* World Health Organization

^a^Haemoglobin, white blood cell, lymphocyte count, platelet count, glucose, blood urea nitrogen, serum creatinine, sodium, potassium, LDH, alkaline phosphatase, albumin, ALT, AST, total bilirubin, CRP, prothrombin time, D-dimer, ferritin level, interleukin-6, and procalcitonin

Safety of the intervention will be actively evaluated by daily interrogation of the following common adverse effects of dexamethasone: hyperglycaemia, steroid-induced psychosis, gastrointestinal bleed, new infection, and chorioretinopathy or blurred vision [25].

### Primary outcome

The primary outcome is a composite of development of moderate or more severe ARDS and all-cause mortality during the 30-day period following enrolment. Based on the Berlin criteria, moderate ARDS is defined by a PaO2/FiO2 > 100 mmHg and ≤ 200 mmHg, and severe ARDS a PaO2/FiO2 < 100 mmHg [26]. To determine the ARDS development, PaO2/FiO2 ratio will be monitored at least once daily. Site investigators will be reporting patient status at 30 days, irrespective of whether the patient continues hospitalized, or is discharged home. If patients are discharged alive from the hospital before day 30, the local investigator will contact the patient or relatives by phone to ensure the status of the patient on day 30 after randomization. Readmission with moderate ARDS and mortality during 30-day after randomization will be detected on the follow-up telephone call programme. Before placing a call, the patient medical record will be reviewed. Electronic health record data related to any inpatient or emergency department revisits within 30 days after discharge will be extracted.

### Secondary outcomes

Secondary outcomes are:

- Requirement of non-invasive ventilation or invasive mechanical ventilation
- Hospital length of stay
- All-cause mortality during the 90-day period following enrolment
- All-cause readmission during the 90-day period following discharge

Local investigators will contact the patient or relatives by phone to ensure the status of the patient on day 90 after randomization. The lead investigator in each site will confirm the recorded 90-day readmission.

The incidence of adverse events and serious adverse events during the study period will be reported.

Results will be communicated at scientific meetings and submitted for publication in peer-reviewed international journals, regardless of the outcome.

### Sample size

We powered the study to assess whether the intervention would reduce the incidence of ARDS. Based on available evidence, we estimate a 30% risk of ARDS in the control group [2]. We are looking for an ARDS 10% absolute reduction in the intervention group. Assuming 80% power and two-sided alpha = 0.05, 226 participants would be needed to detect the resulting reduction of ARDS from 30% in the control group to 20% in the intervention group. We adopted a sample size of 252 (126 per group) to compensate for the potential loss to follow-up of some participants.

### Statistical analysis

Baseline demographic and clinical characteristics will be presented in both groups. Quantitative variables will be defined by mean and standard deviation. Continuous variables will be compared using Student’s *t*-test (normal distribution) or Mann–Whitney test (non-normal distribution). Qualitative variables will be defined by frequency and compared using the chi-squared test or Fisher’s exact test. The proportion of patients that meet the primary outcome will be calculated in the control group and the intervention group. The primary endpoint will be analysed using the chi-squared test comparing the unadjusted risk ratios in each arm. The estimates of risk ratios will be adjusted with multivariate analysis by logistic regression. Adjustment will be performed for age, gender, and history of diabetes, hypertension, or obesity which are the most important factors related to the mortality and disease progression in patients with COVID-19 [27]. Study site will also be included in the multivariate analysis. As a secondary analysis, survival analysis to show the effect of dexamethasone on the primary endpoint in each arm will be performed using Kaplan–Meier curves and log-rank test. A *p*-value of less than 0.05 will be considered statistically significant. Statistical analysis will be performed with the SPSS V28.0 software (SPSS, Inc. Chicago, IL, USA). CONSORT reporting guidelines will be followed to ensure that results are presented in a complete, detailed, and systematic way.

### Data management

All participants will be assigned an identification code to ensure the anonymization of their data. The investigator will maintain a subject identification list for the trial centre (subject identification codes with the corresponding subject names) to enable records to be identified. The sponsor, the trial site, and study staff will handle the subject’s personal and trial data according to the effective legislation regarding data protection. Any paper or electronic trial documents or data are confidential and will not be disclosed to third persons. In the informed consent form, the participants are informed that their medical records will be provided only to authorized monitors, auditors, or inspectors. Data will be collected in each participating centre using an electronic case report form (eCRF) within the REDCap system. The investigator is responsible for the data correctness, completeness, and filling in time. Only the principal investigators and the external Data and Safety Monitoring Board will have access to the trial dataset. External Data and Safety Monitoring Board will assess the trial progress, safety data, and the scientific validity of the results. Medical records of the subjects will be retained in the trial site for 25 years according to the European standards for clinical trials [28].

### Safety assessment

All patients who have been randomized and have taken at least one dose of treatment will be analysed for safety. Safety outcomes include bleeding, metabolic complications (ketoacidosis, hyperglycaemic hyperosmolar state, hyperglycaemia requiring new use of insulin), hypertension, new cardiac arrhythmia, allergic reaction, psychiatric disorders, new infections, and chorioretinopathy or blurred vision. All adverse events occurring after entry into the study and until hospital discharge will be recorded in the patient’s health record. An adverse event that meets the criteria for a serious adverse event between study enrolment and hospital discharge will be reported to the local Institutional Review Board. A serious adverse event for this study is any untoward medical occurrence that is believed by the investigators to be causally related to the study-drug and results in any of the following: life-threatening condition, severe or permanent disability, prolonged hospitalization, or a significant hazard as determined by the Data Safety Monitoring Board. Serious adverse events occurring after a participant is discontinued from the study will not be reported unless the investigators feel that the event may have been caused by the study drug.

## Discussion

Up to one-third of patients with mild COVID-19 pneumonia develop ARDS [13]. In 25% of these patients, ARDS progresses rapidly, requiring urgent care in intermediate respiratory care or intensive care units [29]. Based on initial findings from the first reports from Wuhan, the use of systemic corticosteroids was not recommended in the treatment of patients with COVID-19 pneumonia, even in those at risk of ARDS [30]. However, the RECOVERY trial marked an inflexion point in the use of corticosteroids for the treatment of COVID-19 patients. The benefit was clear in patients with oxygen needs and in those treated more than 7 days after symptom onset, when inflammatory lung damage is likely to have been more common. No benefit in patients who did not require respiratory support was observed, according to the trial’s findings. Due to the elevated risk of progression to ARDS by approximately 30% of patients with COVID-19 pneumonia [2], identification of factors that allow for early identification and treatment of patients who will progress to ARDS is crucial. Inflammatory biomarkers such as LDH, CRP, and lymphocyte are independently associated with increased risk of severe disease [31], so tracking these markers might allow early identification or even prediction of ARDS Unfortunately, the RECOVERY trial did not collect laboratory biomarkers. Although early administration of corticosteroids could blunt the immune response in the initial stage of viral replication, the benefit of dexamethasone in this specific subpopulation of COVID-19 patients with hyperinflammation could be underestimated.

To test this hypothesis, we are conducting a trial to evaluate if the administration of dexamethasone in hospitalized patients with COVID-19 pneumonia who do not require supplementary oxygen and are at risk for severe disease leads to a decrease in the progression to ARDS and death. To our knowledge, this is the first prospective randomized controlled trial to evaluate the benefit of corticosteroids in this subgroup of patients with COVID-19.

Patients receiving glucocorticoids should be monitored for adverse effects. These include hyperglycaemia, an increased risk of infections (including bacterial, fungal, and *Strongyloides* infections) and prolonged viremia. However, in RECOVERY trial record, only four serious adverse events (0.19%) were deemed by the investigators to be related to dexamethasone (two of hyperglycaemia, one of gastrointestinal haemorrhage, and one of psychosis) [15]. Evidence is inconsistent on the viral clearance delay of glucocorticoid-treated COVID-19 patients [32, 33]. Nevertheless, it seems that a high or medium dose, but not a low dose of glucocorticoids, leads to viral clearance delay which seems clear [33]. The rates of infections in patients with COVID-19 receiving glucocorticoids are uncertain. In the same way as viral clearance, prolonged high-dose glucocorticoids increase the risk of the appearance of severe microbial infections [34]. At the proposed dose and period of treatment of dexamethasone in this study, low toxicity and good tolerability profiles are expected.

The major limitation of our study is that it is not blinded and lacks the use of a placebo. However, the primary endpoint of the trial is clear, causing the influence of placebo treatment to be smaller. In addition, with placebo treatment, the trial design would be more complex and the cost more expensive. Second, our study design will not allow us to conclude whether the administration of dexamethasone at different doses and for longer or shorter periods would have improved outcomes. Nevertheless, if our hypothesis is correct, our findings will provide novel insights about the management of patients with COVID-19 pneumonia without additional oxygen requirements and at risk of progressing to severe disease.

### Trial status

Trial status: Recruiting. The first patient was recruited in June 2021. The last patient is anticipated to be recruited in December 2022.

### Supplementary Information

**Additional file 1. **Ethics Committees Approval Document.

**Additional file 2. **AEMPS Approval Document Spanish.

**Additional file 3. **SPIRIT 2013 Checklist: Recommended items to address in a clinical trial protocol and related documents*.

## Data Availability

Only authorized personnel directly involved with the study will have access to the de-identified, coded, computerized database. Participants’ records will be available to study investigators, the European Medicines Agency, the Spanish Agency of Medicines and Medical Devices, the manufacturer of the study product, and the ethics committee.

## Appendix

EARLY-DEX COVID-19 Investigators: Anabel Franco-Moreno, María Soledad Acedo-Gutiérrez, Juan Torres-Macho, Nicolás Labrador-San Martín, Mateo Balado-Rico, Ana Rocío Romero-Paternina, Belén Escolano-Fernández, Esther Piniella-Ruiz, Ester Alonso-Monge, Helena Notario-Leo, Carlos Bibiano-Guillén, Gabriela Peña-Lillo, Armando Antiqueira-Pérez, Jesús Troya-García, María de Carranza-López, Ismael Escobar-Rodríguez (Infanta Leonor–Virgen de la Torre University Hospital); Clara Hernández-Blanco, Rodolfo Romero-Pareja, David Alonso-Menchén, Ana Suárez-Simón (Enfermera Isabel Zendal Emergency Hospital); Noemí Cabello-Clotet, Vicente Estrada-Pérez (Clínico San Carlos University Hospital); Celia Rodríguez-Olleros, Fátima Ibáñez-Estéllez (Puerta de Hierro University Hospital); Nacho Vallejo-Maroto, José Jiménez-Torres (Emergencia COVID-19 Hospital); Ana Castuera-Gil, Juan Antonio Andueza-Lillo (Gregorio Marañón University Hospital).

## Abbreviations

COVID-19: Coronavirus disease 2019
ARDS: Acute respiratory distress syndrome
LDH: Lactate dehydrogenase
CRP: C-reactive protein
SpO2: Peripheral capillary oxygen saturation
PaO2: Partial pressure of arterial oxygen
FiO2: Fraction of inspired oxygen
qSOFA: Quick Sequential Organs Failure Assessment
WHO: World Health Organization
