# Supplementary material for: Effect of EARLY administration of DEXamethasone in patients with COVID-19 pneumonia without acute hypoxemic respiratory failure and risk of development of acute respiratory distress syndrome (EARLY-DEX COVID-19): study protocol for a randomized controlled trial
Source: Trials. 2022 Sep 15;23:784. doi: 10.1186/s13063-022-06722-x (PMC9479245; doi:10.1186/s13063-022-06722-x)
Supplement: Supplementary file 1 — Additional file 1. Ethics Committees Approval Document. [file 13063_2022_6722_MOESM1_ESM.docx]

**This is a true translation from Spanish into English of the Report issued by the Ethics Committee of the key relevant information for the trial protocol on 19 May 2021** (provided by the principal investigator and corresponding author Anabel Franco-Moreno)

Mr. Miguel Ángel Lobo Álvarez, President of the ETHICS COMMITTEE FOR RESEARCH ON DRUGS of the Comunidad de Madrid

CERTIFIES

That this Committee has evaluated the following proposal of a clinical trial

Code: **EARLY-DEX COVID-19** EUDRACT No.: **2021-001028-16**

# Title: Effect of EARLY administration of DEXamethasone in patients with COVID-19 pneumonia without acute hypoxemic respiratory failure and risk of development of acute respiratory distress syndrome (EARLY-DEX COVID-19).

Sponsor Fundación para la Investigación e Innovación Biomédica (FIIB) del Hospital Universitario Infanta Leonor y Hospital Universitario del Sureste.

Protocol: Version 1.2, 17 April 2021

That this Committee has done the evaluation of Part II of the proposal of authorization of the trial, according to the Royal Decree 1090/2015 and the Art 7 of the Regulation 536/2014 of the EU, and considers that:

The procedure to obtain the informed consent (including the information pages for the subject of the trial and the informed consent mentioned in the heading) and the plan and methods about enrollment of patients are adequate and meet the requirements provided in the chapter II of the Royal Decree 1090/2015.

- The compensations to participants are adequate, and the previsions for harms that participants could have.
- The procedure for management of personal data is adequate.
- The future use of biological samples obtained during the trial is adapted to what is provided in the Royal Decree 1716/2011.
- For performing the trial, the participating centers and investigators are adequate, as required in the Annex II to this Report, taking into account the information on adequacy provided by the promotor and by the participating centers.

That this Committee decided to emit a **FAVOURABLE DECISION** in the meeting held on the day 10/05/2021 (Act no. 05/21)

That in that meeting, the requirements required by the current legislation –Royal Decree 1090/2015- were met for making valid the decision of this CEIm.

That the ETHICS COMMITTEE FOR RESEARCH ON DRUGS of the Comunidad de Madrid, its structure and its procedures, meets the norms of Good Clinical Practices (CPMP/ICH/135/95) and the current legislation that regulates its function, and that the structure of the ETHICS COMMITTEE FOR RESEARCH ON DRUGS of the Comunidad de Madrid is in agreement with Annex I, taking into account that in case of any member is involved in the trial or declares a conflict of interest will not participate in the evaluation or neither in the decision of the proposal of authorization of the clinical trial.


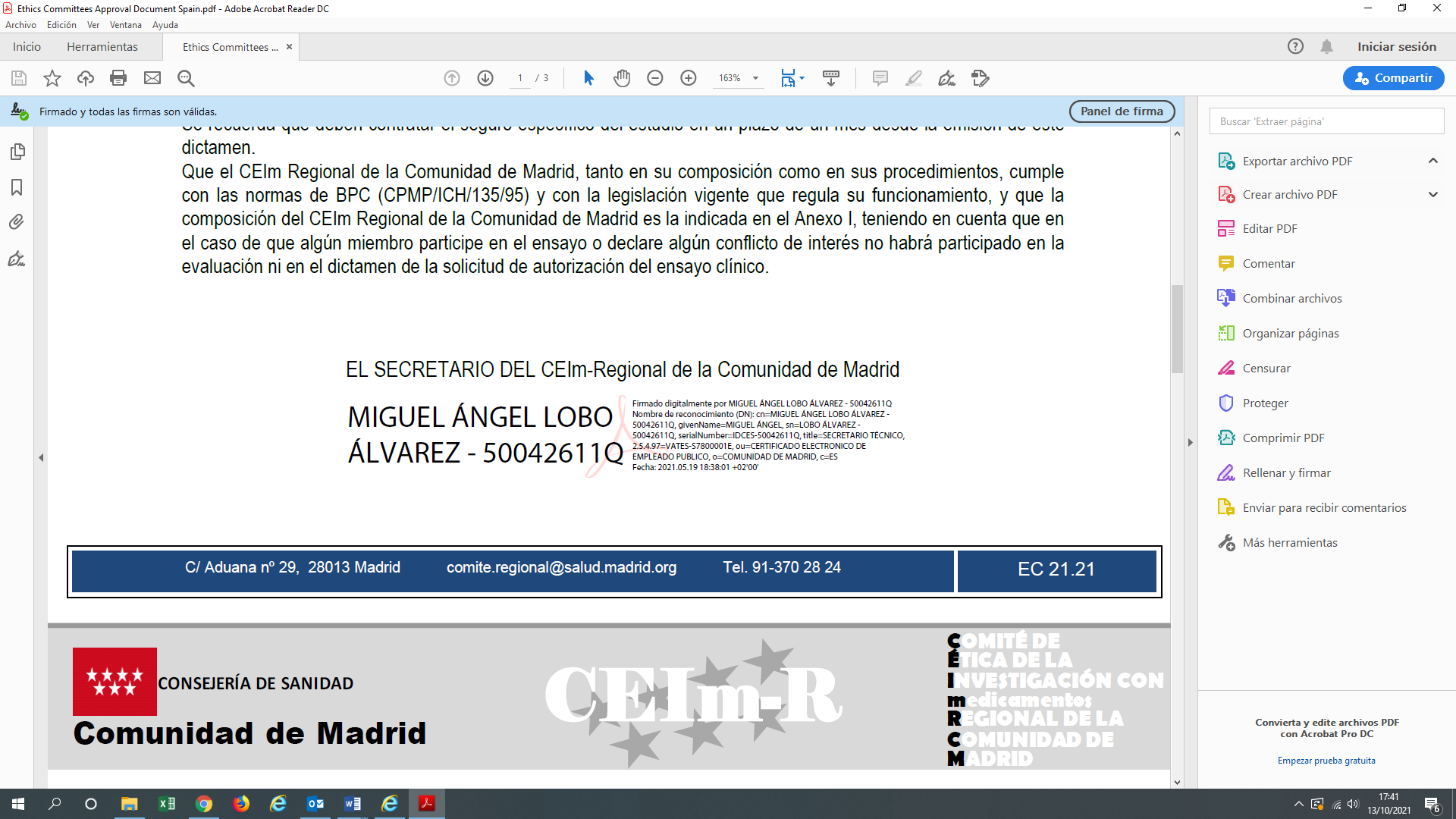


President of the ETHICS COMMITTEE FOR RESEARCH ON DRUGS of the Comunidad de Madrid.
