# Supplementary material for: Effect of EARLY administration of DEXamethasone in patients with COVID-19 pneumonia without acute hypoxemic respiratory failure and risk of development of acute respiratory distress syndrome (EARLY-DEX COVID-19): study protocol for a randomized controlled trial
Source: Trials. 2022 Sep 15;23:784. doi: 10.1186/s13063-022-06722-x (PMC9479245; doi:10.1186/s13063-022-06722-x)
Supplement: Supplementary file 2 — Additional file 2. AEMPS Approval Document Spanish. [file 13063_2022_6722_MOESM2_ESM.pdf]

Referencia: MUH/CLIN/EC

ASUNTO: RESOLUCIÓN DE LA SOLICITUD DE AUTORIZACIÓN DE UN ENSAYO CLÍNICO

DESTINATARIO: Fundación para la Investigación e Innovación Biomédica (FIIB) del Hospital Universitario Infanta Leonor y Hospital Unive
Calle Puerto de Lumbreras, 5
28031 Madrid (España)

DATOS DE LA SOLICITUD

Solicitud de autorizacion del Ensayo clinico Nº EudraCT **2021-001028-16** y título **Administración precoz de dexametasona en pacientes hospitalizados por neumonía Covid-19 y alto riesgo de evolución a síndrome de distrés respiratorio agudo**.

Promotor: Fundación para la Investigación e Innovación Biomédica (FIIB) del Hospital Universitario Infanta Leonor y Hospital Unive
Calle Puerto de Lumbreras, 5
28031 Madrid (España)

Fecha de solicitud válida: 13/03/2021

Una vez evaluada la solicitud de autorización de ensayo clínico previamente indicada, se considera que cumple con los requisitos indicados en el Real Decreto 1090/2015, de 4 de diciembre, por el que se regulan los ensayos clínicos con medicamentos, los Comités de Ética de la Investigación con medicamentos y el Registro Español de Estudios Clínicos y demás legislación aplicable*.

Por todo lo anteriormente expuesto la Directora de la Agencia de Medicamentos y Productos Sanitarios en el ejercicio de sus competencias **RESUELVE:**

AUTORIZAR el ensayo clínico solicitado.

Contra esta Resolución, que pone fin a la vía administrativa, puede interponerse potestativamente Recurso de Reposición ante el/la Director/a de la Agencia Española de Medicamentos y Productos Sanitarios en el plazo de un mes, conforme a lo dispuesto en los artículos 123 y 124 de la Ley 39/2015, de 1 de octubre, del Procedimiento Administrativo Común de las Administraciones Públicas,

* Texto refundido de la Ley de Garantías y Uso Racional de los medicamentos y productos sanitarios, aprobado por Real Decreto Legislativo 1/2015, de 24 de julio.
Real Decreto 1275/2011, de 16 de septiembre, por el que se crea la Agencia estatal "Agencia Española de Medicamentos y Productos Sanitarios y se aprueba su Estatuto".

CORREO ELECTRÓNICO
smhaem@aemps.es
C/ CAMPEZO, 1 - EDIFICIO 8
28022 MADRID
Tel.: 918225073
Fax: 918225043

o interponerse Recurso Contencioso-Administrativo ante el Juzgado Central de lo Contencioso-Administrativo de Madrid, en el plazo de dos meses a contar desde el día siguiente a la recepción de la presente notificación, conforme a lo dispuesto en la Ley Reguladora de la Jurisdicción Contencioso-Administrativa de 13 de julio de 1998, y sin perjuicio de cualquier otro recurso que pudiera interponerse.

DIRECTORA DE LA AGENCIA ESPAÑOLA DE MEDICAMENTOS Y PRODUCTOS SANITARIOS

agencia española de medicamentos y productos sanitarios

Fdo. Mª Jesús Lamas Díaz

CORREO ELECTRÓNICO
smhaem@aemps.es

C/ CAMPEZO, 1 - EDIFICIO 8
28022 MADRID
Tel.: 918225073
Fax: 918225043
