# Supplementary material for: Effect of EARLY administration of DEXamethasone in patients with COVID-19 pneumonia without acute hypoxemic respiratory failure and risk of development of acute respiratory distress syndrome (EARLY-DEX COVID-19): study protocol for a randomized controlled trial
Source: Trials. 2022 Sep 15;23:784. doi: 10.1186/s13063-022-06722-x (PMC9479245; doi:10.1186/s13063-022-06722-x)
Supplement: Supplementary file 3 — Additional file 3. SPIRIT 2013 Checklist: Recommended items to address in a clinical trial protocol and related documents*. [file 13063_2022_6722_MOESM3_ESM.doc]

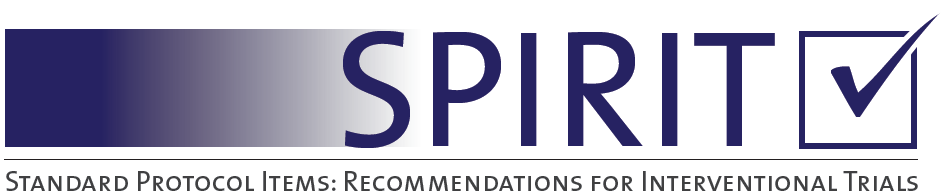


SPIRIT 2013 Checklist: Recommended items to address in a clinical trial protocol and related documents*

**Effect of EARLY administration of DEXamethasone in patients with COVID-19 pneumonia without acute hypoxemic respiratory failure and risk of development of acute respiratory distress syndrome (EARLY-DEX COVID-19)**

| Section/item | ItemNo | Description |
| --- | --- | --- |
| **Administrative information** | | |
| Title | 1 | Effect of EARLY administration of DEXamethasone in patients with COVID-19 pneumonia without acute hypoxemic respiratory failure, and high risk of developing acute respiratory distress syndrome (EARLY-DEX COVID-19) |
| Trial registration | 2a | Registered at ClinicalTrials.gov (NCT04836780): Page 3 manuscript |
|  | 2b | The study was not registered at the WHO registration portal |
| Protocol version | 3 | Version 1.2, 17 April 2021. Ethical approval Act no. 05/21 (page 7 of the manuscript) |
| Funding | 4 | EARLY-DEX COVID-19 is funded by an unrestricted gift from pharmaceutical laboratory Kern Pharma, S.L. with NIF/CIF B58296773 under its Programme Grants for Research Programme: Pages 16 of the manuscript |
| Roles and responsibilities | 5a | Names, affiliations, and roles of contributors: Pages 15, 16 of the manuscript |
|  | 5b | Sponsor: Fundación para la Investigación e Innovación Biomédica del Hospital Universitario Infanta Leonor y Hospital Universitario del Sureste. Gran vía del este, 80 28031, Madrid, Spain. Tax identification number: G88098678 |
|  | 5c | Sponsor and funder have no role in study design; collection, management, analysis, and interpretation of data; writing of the report; decision to submit the report for publication; no ultimate authority over any of these activities |
|  | 5d | **Coordinator Investigator and Principal Investigator**  Preparation of protocol and revisions; Preparation of investigators brochure and Case Report Forms; Recruitment of patients  Publication of study reports; Agreement of final protocol; Reviewing progress of study and if necessary agreeing changes to the protocol and/or investigators brochure to facilitate the smooth running of the study  **Local Investigator**  Publication of study reports; Recruitment of patients and liasing with principle investigator; Reviewing progress of study and if necessary agreeing changes to the protocol and/or investigators brochure to facilitate the smooth running of the study; Randomisation  **Sponsor**  Managing Clinical Trials Office; Organising steering committee meetings; Reviewing progress of study and if necessary agreeing changes to the protocol and/or investigators brochure to facilitate the smooth running of the study; Provide annual risk report Medicines and Healthcare products Regulatory Agency and ethics committee; Audit of 3 monthly  **Steering committee** (see Appendix 1 for members: Page 13 of the manuscript)  Agreement of final protocol  **Organisation of steering committee meetings**  Serious unexpected suspected adverse events reporting to Medicines and Healthcare products Regulatory Agency and Kern Pharma  **Data Manager**  Maintenance of trial IT system and data entry; Data verification  **Lead Investigators**: In each participating centre, a lead investigator will be identified, to be responsible for identification, recruitment, data collection and completion of CRFs, along with follow up of study patients and adherence to study protocol and investigators brochure |
| Introduction |  |  |
| Background and rationale | 6a | Pages 4, 5, 6 of the manuscript |
|  | 6b | Page 5 of the manuscript |
| Objectives | 7 | Specific objectives or hypotheses: Page 6 of the manuscript |
| Trial design | 8 | Pages 5, 6 of the manuscript |
| Methods: Participants, interventions, and outcomes | | |
| Study setting | 9 | Page 6 of the manuscript, and Appendix 1 |
| Eligibility criteria | 10 | Page 6 of the manuscript |
| Interventions | 11a | Pages 7, 8 of the manuscript |
|  | 11b | Page 9 of the manuscript |
|  | 11c | Page 9 of the manuscript |
|  | 11d | There are no specific concomitant care and interventions that are permitted or prohibited that are applicable to this trial: Page 9 of manuscript |
| Outcomes | 12 | Pages 10, 11 of the manuscript |
| Participant timeline | 13 | Page 9 of the manuscript. A schematic diagram is provided as Figure 1 |
| Sample size | 14 | Page 11 of the manuscript |
| Recruitment | 15 | Each clinical center involved in the trial was chosen based on provided documentation for patient availability |
| **Methods: Assignment of interventions (for controlled trials)** | | |
| Allocation: |  |  |
| Sequence generation | 16a | Page 8 of the manuscript |
| Allocation concealment mechanism | 16b | Page 8 of the manuscript |
| Implementation | 16c | Pages 8, 9 of the manuscript |
| Blinding (masking) | 17a | No applicable. The trial is not blinded |
|  |  |  |
| **Methods: Data collection, management, and analysis** | | |
| Data collection methods | 18a | Data will be collected in each participating using a electronic case report form (eCRF) within the REDCap system. Pages 12, 13 of the manuscript |
|  | 18b | Responsibility of study coordinator |
| Data management | 19 | Pages 12, 13 of the manuscript |
| Statistical methods | 20a | Page 12 of the manuscript |
|  | 20b | The estimates of rate and risk ratios will be adjusted for the age in three categories (<70 years, 70 to 79 years, and ≥80 years): Page 12 of the manuscript |
|  | 20c | Analysis population: Not applicable; no other analysis populations are planned |
| **Methods: Monitoring** | | |
| Data monitoring | 21a | A Data Monitoring Committee (DMC), independent of the study organisers, will assessment a periodic inspection of the accumulating outcome data by study |
|  | 21b | During the period of recruitment to the study, interim analyses will be supplied, in strict confidence to the DMC |
| Harms | 22 | Pages 10, 11, 13 of the manuscript |
| Auditing | 23 | Not applicable. There is no on-site auditing of the trial. Before exporting the data into a computerized database at the data coordinating center, local investigators will check the completeness and the quality of information |
| Ethics and dissemination | | |
| Research ethics approval | 24 | Page 17 of the manuscript |
| Protocol amendments | 25 | The Steering Committee will communicate substantial protocol modifications (when applicable) to relevant parties (investigators, referral Ethics Committee, Spanish Agency for Drugs, trial participants, regulators) |
| Consent or assent | 26a | Page 7 of the manuscript |
|  | 26b | Additional consent provisions for collection and use of participant data and biological specimens in ancillary studies: Not applicable |
| Confidentiality | 27 | Patient information is anonymized: pages 12, 13 of the manuscript |
| Declaration of interests | 28 | Pages 17, 18 of the manuscript |
| Access to data | 29 | Page 13 of the manuscript |
| Ancillary and post-trial care | 30 | The study include cover for additional health care, compensation or damages whether awarded voluntarily by the Sponsor, or by claims pursued through the courts. Clinical Trial Policy Number: 76358045-30016 |
| Dissemination policy | 31a | Results of the trial will be published in peer-reviewed international journals, regardless of the outcome. There is no obligation for communicating the results to individual patients |
|  | 31b | All investigators have the right to be authors of the final publication |
|  | 31c | No later than 6 months after the randomisation of the first participant, we will grant public access to the full protocol trial, and no later than 1 year after the randomisation of the last particiant, we will deliver a completely deidentified data set to an appropriate data archive for sharing purposes |
| Appendices |  |  |
| Informed consent materials | 32 | The model of the informed consent (in Spanish) is provided as the Additional File 5 |
| Biological specimens | 33 | Not applicable |

*It is strongly recommended that this checklist be read in conjunction with the SPIRIT 2013 Explanation & Elaboration for important clarification on the items. Amendments to the protocol should be tracked and dated. The SPIRIT checklist is copyrighted by the SPIRIT Group under the Creative Commons “[Attribution-NonCommercial-NoDerivs 3.0 Unported](http://www.creativecommons.org/licenses/by-nc-nd/3.0/)” license.
